# Comparison between Two Different Synthesis Methods of Strontium-Doped Hydroxyapatite Designed for Osteoporotic Bone Restoration

**DOI:** 10.3390/ma17071472

**Published:** 2024-03-23

**Authors:** Cosmin Iulian Codrea, Daniel Lincu, Irina Atkinson, Daniela C. Culita, Alexa-Maria Croitoru, Georgiana Dolete, Roxana Trusca, Bogdan Stefan Vasile, Miruna Silvia Stan, Denisa Ficai, Anton Ficai

**Affiliations:** 1 Faculty of Chemical Engineering and Biotechnologies, National University of Science and Technology Politehnica of Bucharest, 060042 Bucharest, Romania; daniel.lincu1113a@gmail.com (D.L.); alexa_maria.croitoru@upb.ro (A.-M.C.); dolete.georgiana@gmail.com (G.D.); truscaroxana@yahoo.com (R.T.); bogdan.vasile@upb.ro (B.S.V.); denisaficai@yahoo.ro (D.F.); 2 Department of Oxide Compounds and Materials Science, Institute of Physical Chemistry “Ilie Murgulescu” of the Romanian Academy, 060021 Bucharest, Romania; iatkinson@icf.ro (I.A.);; 3 National Research Center for Micro and Nanomaterials, Faculty of Chemical Engineering and Biotechnologies, National University of Science and Technology Politehnica of Bucharest, Splaiul Independentei 313, 060042 Bucharest, Romania; 4 National Centre for Food Safety, National University of Science and Technology Politehnica of Bucharest, Splaiul Independentei 313, 060042 Bucharest, Romania; 5 Department of Biochemistry and Molecular Biology, Faculty of Biology, University of Bucharest, Splaiul Independentei 91-95, 050095 Bucharest, Romania; miruna.stan@bio.unibuc.ro; 6 Academy of Romanian Scientists, Ilfov St. 3, 050044 Bucharest, Romania

**Keywords:** hydroxyapatite nano-powder, co-precipitation, hydrothermal, strontium

## Abstract

Development of efficient controlled local release of drugs that prevent systemic side effects is a challenge for anti-osteoporotic treatments. Research for new bone-regeneration materials is of high importance. Strontium (Sr) is known as an anti-resorptive and anabolic agent useful in treating osteoporosis. In this study, we compared two different types of synthesis used for obtaining nano hydroxyapatite (HA) and Sr-containing nano hydroxyapatite (SrHA) for bone tissue engineering. Synthesis of HA and SrHA was performed using co-precipitation and hydrothermal methods. Regardless of the synthesis route for the SrHA, the intended content of Sr was 1, 5, 10, 20, and 30 molar %. The chemical, morphological, and biocompatibility properties of HA and SrHA were investigated. Based on our results, it was shown that HA and SrHA exhibited low cytotoxicity and demonstrated toxic behavior only at higher Sr concentrations.

## 1. Introduction

The clinical as well as the economic impact for the treatment of bone diseases is remarkable, bone being one of the most transplanted tissues and accounting for almost 50% of the total need of implants. Significant ongoing demand has led researchers to look for osteogenic biomaterials that are efficacious, convenient for surgeons, cost-effective, and easy to be manufactured at the scales required. Osteoporosis is the cause of a huge financial cost and is expected to rise as the percentage of the aging population increases, as mentioned by numerous authors [1,2,3,4,5,6]. Biological apatite is one of the main constituents of bone tissue along with type I collagen and several other non-collagenous proteins [7]. Hydroxyapatite (HA), which is very similar to biological apatite [8], is a promising bone substitute mineral and still attracts great interest. HA is a type of calcium phosphate (CaP) extensively used in implants or coatings for biomedical applications due to its biocompatibility [9] and well-known osteoconductive [10] and osteoinductive properties [8]. These advantages enable HA to be used also as drug/protein/gene loading in delivery systems, and potentially in cell targeting, fluorescence labeling, and imaging and diagnosis materials, and is a model compound that mimics the biomineralization process [11]. In addition, nanosized three-dimensional multiporous CaPs show fascinating tissue-induced bone regeneration activity [12]. Mechanical stiffness characteristics of HA that contribute to its osteogenic bioactivity makes also surgical implementation challenging, as porous HA cannot be easily shaped and resized by surgeons on demand to better accommodate the defect site [13].

The biological HA crystals in the hard tissues of organisms exist in either a needle-like or plate-like form with thicknesses of several nanometers and lengths of tens of nanometers [12]. Nanoparticles (NPs) with lower crystallinity resemble more the physico-chemical properties of bone mineral [14], as apatite has poor crystallinity, is nonstoichiometric and contains carbonate as well as other cation/anion substitutions [15,16]. HA NPs exhibit better bioactivity and higher resorbability than particles in microscale sizes [11]. Crystalline HA is known to be resorbed very slowly in vivo [15]. Mixtures of CaP phases have greater solubility in vivo than pure HA, hence, scaffold implants based on materials with less purity and crystalline should be resorbed more rapidly [15]. Also, calcium-deficient HA NPs with elongated needle-like structure could better facilitate osteogenesis of bone-forming cells [17]. Solubility of HA (the second most thermodynamically stable CaP close to fluorapatite), is a complex process, controlled by various factors (pH, ionic strength, Ca/P ratio, NP size, structural defects, and ionic substitutions). Apatite dissolution occurs through the formation of pits on the crystal surface which influence the dissolution rate. These findings suggest that size and defects of apatite play an active role [18]. Bone tissue is a complex composite material which can be described as an apatite matrix reinforced with mineralized collagen fibers where the apatitic material is substituted with several cations, such as Si^4+^, Mg^2+^, and Zn^2+^, but also with anions, especially carbonate and citrate. Hence, introducing different cations into the HA lattice is a commonly used method for improving implant performances. Strontium (Sr) is a trace element found in the human body, 98% of which is found in the skeleton [19], and which can improve new bone formation [17].

Cells are influenced by surface chemistry, roughness, and surface-free energy, through the effects of these proprieties on protein adsorption. Rough surfaces may physically trap fibrin molecules and localize signaling molecules near the surface of an implant, and it is thought that osteoprogenitor cells may be led by the fibrin matrix toward the implant surface as fibrinolysis occurs. Nanoscale topography of CaP ceramics enhances osteoblast adhesion and function, decreases fibroblast adhesion, and enhances bone remodeling [20].

In this extensive study, we compared co-precipitation and different hydrothermal synthesis routes for the HA/Sr-containing nano hydroxyapatite (SrHA), as well as the effect of different Sr^2+^ concentrations regarding the morphological and the biological properties of the obtained materials. The two chosen synthesis routes do not make use of the sintering step, which is prone to formation of secondary phases [21], making it an easy and more cost-effective way of obtaining the powder material. The presence of Sr^2+^ is expected not to increase the cytotoxic effect, at least at lower concentrations. For a more accurate estimation of the Sr^2+^ presence effect, we used a wide range of intended Sr content, more precisely Sr/(Ca + Sr) molar ratios of 1, 5, 10, 20, and 30%.

Sr ions released directly from the synthetic bone substitute during its resorption could potentially address the challenges associated with incorporating Sr into bone following oral administration [22]. The comparison made in this study may be used to determine the best variant of HA/SrHA for incorporating in new composites that better match bone-tissue-specific needs and, thus, develop more complex bone substitutes. Our extensive study is notable because it is one of the few to investigate a wider concentration range, while comparing two different synthesis methods.

## 2. Materials and Methods

### 2.1. Synthesis of HA and SrHA Powders

Calcium nitrate tetrahydrate Ca(NO_3_)_2_·4H_2_O, Sigma, Burlington, MA, USA, 2322231, strontium nitrate Sr(NO_3_)_2_ p.a. > 99%, Fluka, Buchs, Switzerland, 85899, and diammonium hydrogen phosphate (NH_4_)_2_HPO_4_, 98+%, Aldrich, Burlington, MA, USA, 215996, were used for the synthesis of HA and SrHA. We started with calcium nitrate tetrahydrate Ca(NO_3_)_2_·4H_2_O for HA and added various amounts of strontium nitrate Sr(NO_3_)_2_ as the Sr source in order to obtain SrHA. Subsequently, we added dropwise diammonium hydrogen phosphate (NH_4_)_2_HPO_4_ for about 30 min at room temperature, under continuous magnetic stirring, while the pH was kept above 10 by adding ammonium hydroxide NH_4_OH, Silal Trading, Bucharest, Romania, 2156476. HA/SrHA was immediately formed and observed as a white precipitate. The concentrations used were intended to respect the natural bone ratio of (Ca + Sr)/P of 1.67.

For the simple chemical precipitation wet method, the homogeneous suspension obtained was further stirred for 72 h at room temperature. For the hydrothermal method synthesis, the homogeneous suspension obtained was stirred for 10 min at room temperature and we applied further hydrothermal treatment at 150 °C for 6 h by using a high-temperature and high-pressure airtight vessel (autoclave). The molar ratios of Sr/(Ca + Sr) were intended to be 0, 1, 5, 10, 20, and 30%, and these samples are referred to as HAPR, HAPR-Sr1%, HAPR-Sr5%, HAPR-Sr10%, HAPR-Sr20%, and HAPR-Sr30%, for the precipitation method, respectively, and HAHT, HAHT-Sr1%, HAHT-Sr5%, HAHT-Sr10%, HAHT-Sr20%, and HAHT-Sr30%, for the hydrothermal method, respectively. After 72 h of maturation, and hydrothermal treatment for half of the samples, we subjected both batches of samples to multiple cycles of centrifugation (using a Thermo Scientific, Waltham, MA, USA, centrifuge at 25 °C, 6000 RPM for 10 min), washed them with distilled water, and sonicated the mixture for 10 min. This process aimed to lower the pH and facilitate the separation of the resulting HA/SrHA powders from water. In the end, the obtained HA/SrHA powders were dried at 70 °C for 21 h and crushed with an agate mortar and pestle.

### 2.2. Characterization Methods of HA/SrHA Powders

#### 2.2.1. General Overview of Characterization Methods

The obtained HA/SrHA powders were characterized using several methods in order to evaluate their suitability for bone tissue regeneration. Characterization methods were selected to evaluate the specific properties of the materials. X-ray diffraction (XRD) was used to determine the crystalline phase profile of the synthesized powders. Fourier transform infrared spectroscopy (FTIR) was used to analyze the chemical composition of the powders by detecting the presence of various functional groups and chemical bonds in the material and to determine the HA/SrHA-specific signals. Scanning electron microscopy (SEM) was used to analyze the morphology of the powders and provide high-resolution images that can reveal the structural details of the scaffold surface. Energy-dispersive X-ray (EDX) analysis was used to identify and quantify the elemental composition of a material. The specific surface area of solids by gas adsorption was calculated by the Brunauer–Emmett–Teller (BET) equation in order to determine the specific surface area of a material. Inductively coupled plasma atomic mass spectrometry (ICP-MS) was used in order to examine the release of strontium from our samples in a phosphate buffer solution (PBS) at defined intervals of 1, 3, 7, 14, 21, and 28 days. Cytotoxicity studies were used to evaluate the powder’s biocompatibility.

#### 2.2.2. X-ray Powder Diffraction Analysis

XRD analysis was performed using a Rigaku Ultima IV diffractometer with a monochromatic Cu Kα (*λ* = 1.5418 Å) radiation source, operated at 40 kV and 30 mA. The XRD patterns were recorded in a 5–85° 2θ range, with a scan speed of 2° min^−1^ and a step width of 0.02°. The collected data were analyzed using Rigaku’s PDXL software, version 1.8, Tokyo, Japan, connected to the ICDD PDF-2 database. The lattice parameters were refined using the whole-powder-pattern fitting method (WPPF). The quality of the refinement was evaluated using the goodness of fit S, which should be close to 1, and Rwp (weighted differences between measured and calculated values), which should be close to 10%.

Crystallite size was estimated using Scherrer’s equation from the broadening of (002) reflection, because this plan corresponds to the c crystallographic axis and does not exhibit any interference:

(1) $$D=\frac{k\lambda }{\beta cos\Theta }$$

where *k* is the shape factor taken as 0.90, *β* is full width at half maximum of the intensity, *λ* is wavelength of the Cu Kα radiation (1.54056 Å), and *θ* is Bragg’s diffraction angle.

The crystallinity index (*X_c_*), corresponding to the fraction of the crystalline phase present in the sample, was estimated by an empirical formula as follows [23,24]:

(2) $$Xc={\left(\frac{K}{\beta (002)}\right)}^{3}$$

where *K* is a constant (0.24) and *β* (002) is the full width at half minimum of (002) diffraction line (in degrees).

#### 2.2.3. Fourier Transform Infrared Spectroscopy (FTIR) Analysis

The chemical behavior (homogeneity, degradability, phase interaction) of the scaffolds were examined by FTIR analysis (Nicolet iS50 FT-IR spectrometer, Thermo Fisher Scientific, Waltham, MA, USA). The equipment provides information of high sensitivity between 4000 ${cm}^{-1}$ and 400 ${cm}^{-1}$ at a resolution of 4 ${cm}^{-1}$. The system is equipped with a deuterated triglycine sulfate (DTGS) detector and fitted with a mounted attenuated total reflection (ATR) device. Nicolet Omnic software (version 9.3.32) was used for data analysis.

#### 2.2.4. Scanning Electron Microscopy (SEM) and Energy Dispersive X-ray (EDX) Analysis

SEM and EDX measurements were performed using a field emission gun scanning electron microscope (FEG-SEM) Quanta Inspect F50, FEI, Hillsboro, OR, USA, with a resolution of 1.2 nm, equipped with an EDX module, working at a MnKα resolution of 133 eV. The surface morphologies of the scaffolds as well as the size of the fibers were observed with the same equipment.

#### 2.2.5. Transmission Electron Microscopy (TEM) Analysis

TEM images were obtained for HA/SrHA dried powder samples using a TecnaiTM G2 F30 S-TWIN high-resolution transmission electron microscope from FEI, Hillsboro, OR, USA, operated at an acceleration voltage of 300 kV and equipped with a selected-area electron diffraction (SAED) detector.

#### 2.2.6. Textural Analysis

N_2_ physisorption analyses were conducted at −196 °C using a Micromeritics ASAP 2020 analyzer (Norcross, GA, USA). Before analysis, the samples underwent degassing at 250 °C for 4 h under vacuum. The specific surface areas (S_BET_) were determined using the Brunauer–Emmett–Teller (BET) equation, while the total pore volume was estimated based on the adsorbed amount at the relative pressure of 0.99. Additionally, pore size distribution (PSD) curves were generated from the desorption data employing the Barrett–Joyner–Halenda (BJH) model.

#### 2.2.7. Strontium-Ion Release by Inductively Coupled Plasma Mass Spectrometry (ICP-MS)

The study was performed by dispersing 300 mg of each powder in 200 mL of phosphate buffer solution. Subsequently, the samples were subjected to continuous stirring at a controlled temperature of 37 °C for a period of 28 days. At the previously mentioned time intervals (1, 3, 7, 14, 21, 28 days), a volume of 1 mL of phosphate-buffered saline (PBS) was removed and replaced with fresh medium. This method used for estimating the cumulative release at different time points is referred to as fractional volume sampling and is essential to prevent underestimation of the targeted analyte release. To calculate cumulative Sr release using fractional volume sampling, Equations (3) and (4) were used:

(3) $$Q_{i}=\frac{V_{i}}{V_{T}}\cdot C_{i}\;[\mathrm{mg}],$$

where *Q_i_* is the cumulative Sr release at time point *i*, *V_i_* is the volume of the sample taken at time point *i* (1 mL), *V_T_* is the total volume of the release medium (200 mL), and *C_i_* is the concentration of Sr in the sample at time point *i.*

In order to get the cumulative Sr release up to a certain time point, it is necessary to add up the data corresponding to all preceding time points:

(4) $$\mathrm{Total}\;\mathrm{cumulative}\;\mathrm{release}\;\mathrm{at}\;\mathrm{time}\;t_{i}=\sum_{j=1}^{i}Q_{j}\;[\mathrm{mg}]$$

The removed aliquots were subsequently diluted 10-fold and further analyzed using an Agilent 8800 Triple Quadrupole ICP-MS (Agilent Technologies, Tokyo, Japan), equipped with an ASX500 autosampler, a MicroMist concentric nebulizer, a Peltier cooling spray-chamber set at 2 °C, a torch with an internal diameter of 2.5 mm and Ni sampler, and skimmer cones. The equipment was adjusted in accordance with the specifications provided by the manufacturer and calibrated using a set of five calibration standards having a concentration range between 5 and 100 µg/L Sr. The calibration curve for Sr exhibited a high degree of linearity, as evidenced by the correlation coefficient (R^2^) of 0.9999.

Furthermore, recovery (R) at each time period was calculated using Equation (5):

(5) $$R_{i}=\frac{Q_{i}}{\mathrm{Maximum}\;\mathrm{amount}\;\mathrm{available}}100\;[\%],$$

where the maximum amount available was calculated based on the weight percentage for Sr derived from EDX analysis.

#### 2.2.8. In Vitro Biological Evaluation

Cell culture. Mouse pre-osteoblasts (MC3T3-E1 cell line) were grown in Dulbecco Modified Eagle’s Medium (Invitrogen, Waltham, MA, USA) with 10% fetal bovine serum (Gibco, Waltham, MA, USA) at 37 °C in a humidified atmosphere with 5% CO_2_. The cells were seeded at a cell density of 6 × 10^4^ cells/cm^2^ on the tissue culture plastic surface (TCPS) which served as a control, in the presence of different concentrations of HA/SrHA samples (0, 10, 50, 100, 250, and 500 µg/mL). Biocompatibility tests were executed after 24 h of incubation in standard conditions.

MTT assay. The cellular viability was measured using the 3-(4,5-dimethylthiazol-2-yl)-2,5-diphenyltetrazolium bromide (MTT; Sigma-Aldrich, Burlington, MA, USA) assay. The culture medium was removed at the end of incubation time and the cells were incubated with 1 mg/mL MTT solution for 2 h at 37 °C. The purple formazan crystals formed in the viable cells were dissolved with 2-propanol (Sigma-Aldrich, USA), and the absorbance was measured at 595 nm using a microplate reader (FlexStation 3, Molecular Devices, San Jose, CA, USA).

Nitric oxide (NO) assay. The concentration of NO in the culture medium collected after the 24 h of incubation was measured using the Griess reagent, a stoichiometric solution (*v*/*v*) of 0.1% naphthylethylendiamine dihydrochloride, 1% sulfanilamide in 5% phosphoric acid. Increased levels of NO are related to cytotoxic effects through inflammation and apoptosis. The absorbance of the mix formed by equal volumes of medium supernatants and Griess reagent was read at 550 nm using a microplate reader, and the NO concentration was calculated from the NaNO_2_ standard curve.

Statistical analysis. The in vitro assays were performed in triplicates and the results were calculated as mean ± standard deviation (SD) of three independent experiments. The statistical analysis was carried out on three replicates per sample by unpaired Student’s *t*-tests, and differences were considered significant at *p* values less than 0.05.

## 3. Results and Discussion

### 3.1. X-ray Powder Diffraction

**Figure 1:**
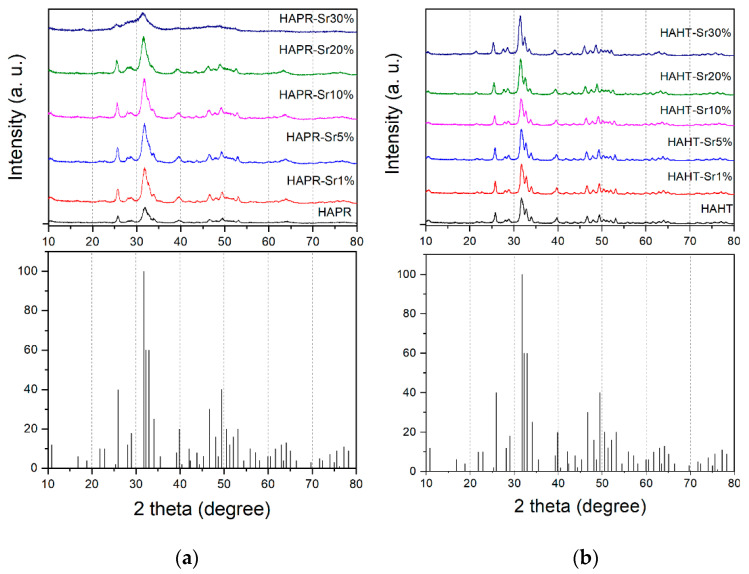
X-ray diffraction (XRD) patterns of pure and strontium-doped hydroxyapatite (HA) obtained via the precipitation method (**a**) and the hydrothermal method (**b**).

XRD patterns of HA/SrHA obtained by wet chemical precipitation and hydrothermal methods are shown in Figure 1. The XRD patterns of the HA/SrHA samples exhibit diffraction peaks that correspond to (211), (112), and (300) reflections of HA [15], in good agreement with Joint Committee on Powder Diffraction Standards (JCPDS) card no. 01-072-1243. Moreover, broad diffraction peaks were observed, indicating the synthesis of low-crystalline HA. No diffraction peaks associated with secondary phases of CaP or Sr compounds were detected, indicating that both synthesis methods employed in this study are suitable for obtaining a stable apatite phase. The absence of additional diffraction peaks attributed to Sr compounds suggests a possible substitution of Sr ions in the HA lattice.

The lattice parameters, the unit cell volumes, and the crystallite size of the obtained HA/SrHA samples calculated from the XRD data are summarized in Table 1. The crystallite size is in the range of 79 to 205 Å. As can be observed, the samples prepared by the wet chemical precipitation method without further thermal treatment had lower crystallinity and smaller crystallite size when compared with the HA obtained by the hydrothermal method. The crystallite size of HA relates to its resorption and solubility. A smaller crystallite size can be an advantage for further applications because it accounts for higher solubility and resorbability of HA in biological environments [25,26].

The calculated lattice parameters and unit cell volume of the samples agree well with the values on the JCPDS card no. 01-072-1243 and increased with the Sr concentration due to the larger Sr^2+^ ionic radius (1.12 Å) compared to the ionic radius of Ca^2+^ (0.99 Å) [2,27]. Moreover, a slight shifting of the diffraction lines’ positions towards lower values is observed due to the replacement of Ca by Sr ions in the HA lattice [28].

### 3.2. Fourier Transform Infrared Spectroscopy (FTIR) Analysis

**Figure 2:**
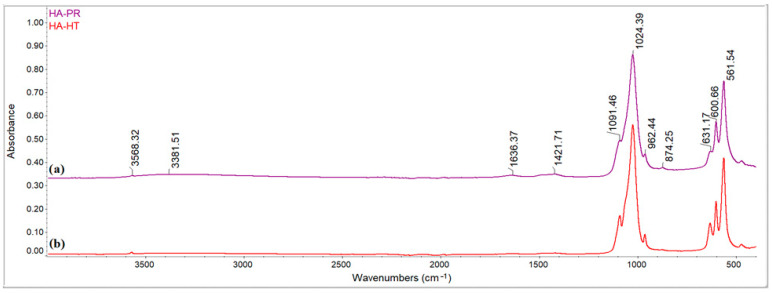
Fourier transform infrared spectroscopy (FTIR) spectra of HA-PR and HA-HT obtained by precipitation (a) and by the hydrothermal method (b).

As represented in Figure 2, FTIR spectra of HA-PR and HA-HT shows the characteristic functional groups of the samples (PO_4_^3−^, HPO_4_^2−^ and CO_3_^2−^). The signals at 561 and 600 cm^−1^ as well as the signals from 962, 1024, and 1091 cm^−1^ are assigned to the PO_4_^3−^ asymmetric bending and asymmetric stretching vibration groups [29]. The stretching vibration of the CO_3_^2−^ from HA samples is observed at 1421 cm^−1^, specific to the substitution of B-type HA with a low unit cell volume [30], and the signal at 874 cm^−1^ can indicate the presence of the CO_3_^2−^ ions, which might occur during the synthesis [31]. The absence of the 1550 cm^−1^ vibration points out the low replacement of OH^−^ groups by CO_3_^2−^ in the channels of the HA structure and thus, the absence of A-type HA [30]. The signal at 1636 cm^−1^ could be assigned to the in-plane H–O–H bending mode of the absorbed water. The signal at 3381 cm^−1^ and the weak peak at 631 cm^−1^ correspond to the OH^−^ bending vibration modes. Also, the signal composed of a shoulder around 3500 cm^−1^ is associated with the OH^−^ bands of HA samples [32,33].

FTIR spectra show clear differences between the two methods used for obtaining HA. Using the hydrothermal technique, an increase in the relative intensity and sharpness of HA peaks can be noticed, indicating a more highly crystallized HA phase (Figure 2b). In contrast, HA obtained by precipitation technique shows relatively broad peaks in the FTIR spectrum, due to the poorly crystalline apatite phases (Figure 2a). The obtained FTIR results strengthen the findings of XRD analysis, confirming the HA structure of the material.

**Figure 3:**
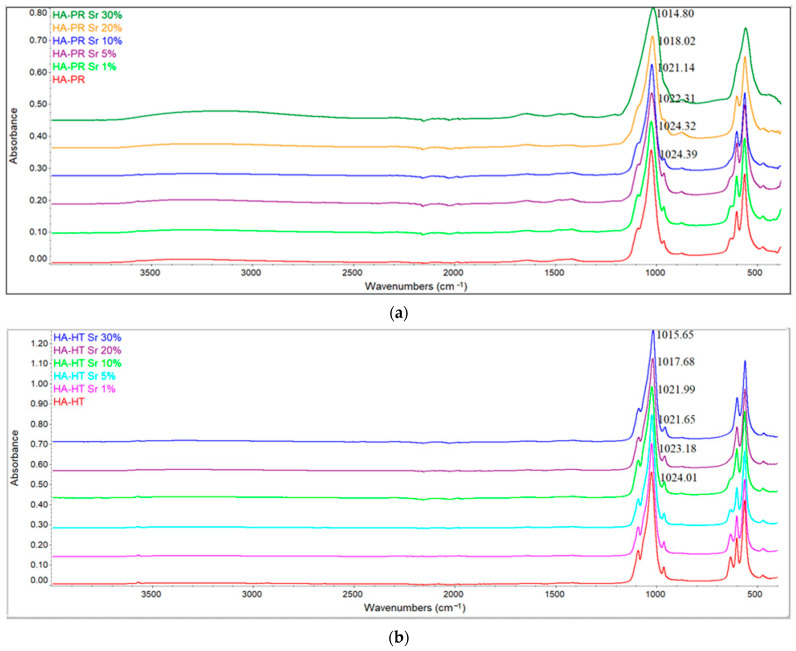
FTIR spectra of SrHA obtained by precipitation (**a**) and by the hydrothermal method (**b**).

The FTIR spectra of HA-PR and HA-HT doped with different concentrations of Sr are presented in Figure 3. After Sr substitution, all specific bands of HA can still be noticed for all prepared samples. Nevertheless, a decrease in the relative intensity of the peaks around 630 cm^−1^ (attributed to OH^−^ bending vibration modes), can be observed due to the increase in the Sr concentration. Partial replacement of Ca^2+^ ions by Sr^2+^ ions causes a crystal lattice expansion due to the larger atomic radius of the Sr element [34]. This determines a smaller crystallite size for the powders and the incorporation of CO_3_^2−^ ions in HA structure, and, thus, a decreased intensity of the above-mentioned peaks [35].

Furthermore, the intensity of the bands around 960 and 1090 cm^−1^, assigned to PO_4_^3−^, are also decreasing. Loss in the resolution and broadening of the PO_4_^3−^ bands is related to a decrease in crystallinity of SrHA [36], due to the enhancement of the Sr concentrations, confirming the substitution of Ca^2+^ ions by larger-sized Sr^2+^ ions. In the PR method, a visible shifting and broadening of the peak between 980 and 1080 cm^−1^ can be observed, also highlighting the incorporation of Sr within HA crystal lattice and, thus, a decrease in crystallinity [37], similar to the findings of XRD analysis. The same shifting can be observed in the HT method.

**Figure 4:**
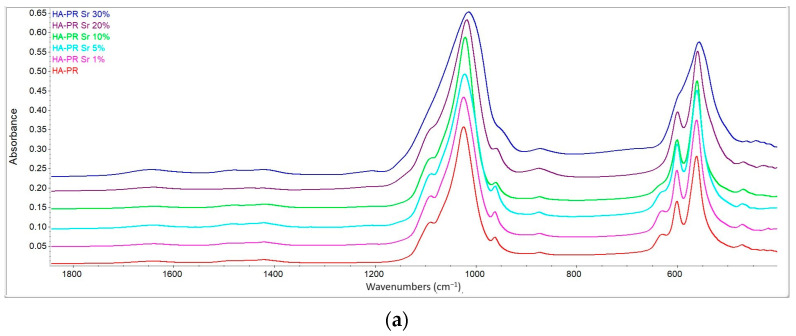

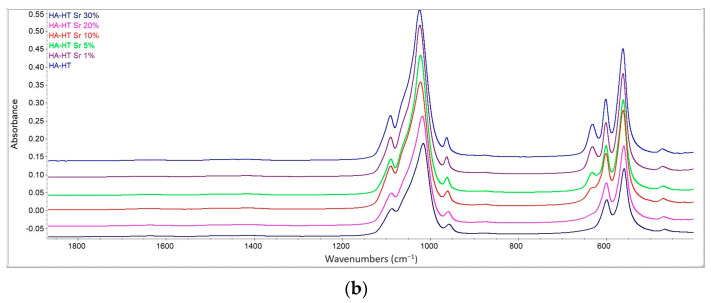
Detailed FTIR spectra of HA/SrHA obtained by precipitation (**a**) and by the hydrothermal method (**b**).

It can be concluded that FTIR results show no significant changes in the HA peaks after Sr substitution and that the samples were HA crystals [27,33]. Also, the intensity of the peaks is higher in samples obtained by the HT method, compared to samples obtained by the PR method (Figure 4), due to higher crystallinity and less substitution of HA-specific chemical groups.

### 3.3. Textural Analysis

**Figure 5:**
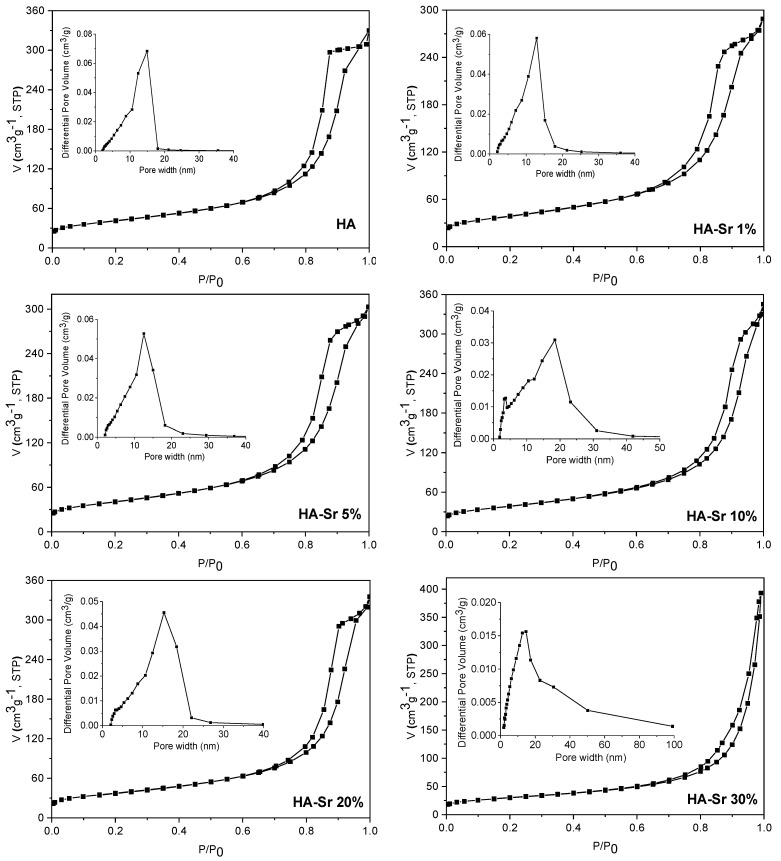
N_2_ adsorption–desorption isotherms and pore size distribution (inset) of the samples: HA; HA-Sr 1%; HA-Sr 5%; HA-Sr 10%; HA-Sr 20%; HA-Sr 30%.

**Figure 6:**
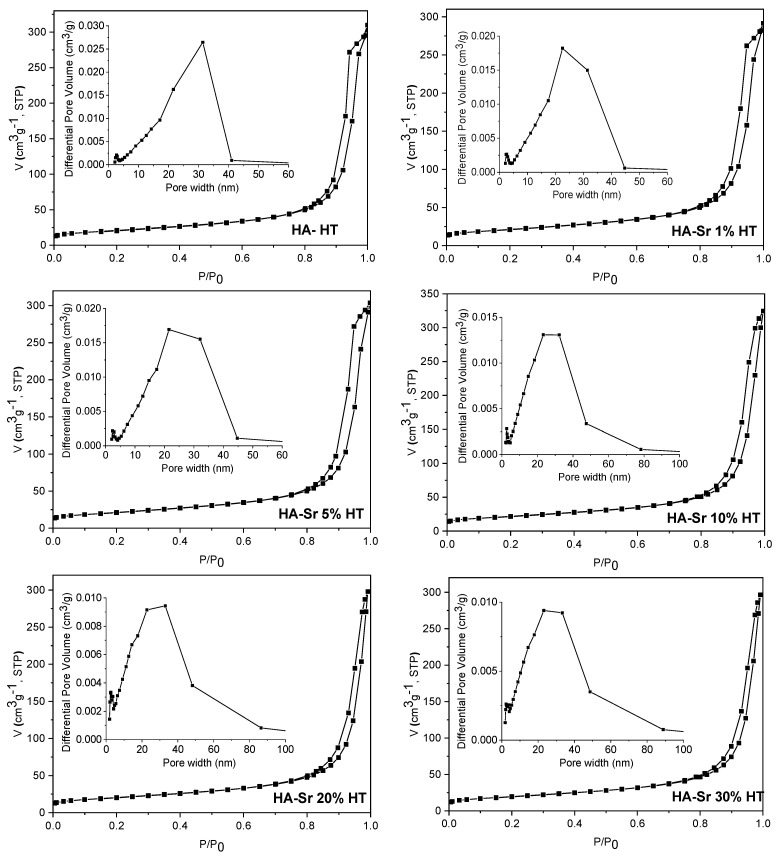
N_2_ adsorption–desorption isotherms and pore size distribution (inset) of the samples: HA HT; HA-Sr 1% HT; HA-Sr 5% HT; HA-Sr 10% HT; HA-Sr 20% HT; HA-Sr 30% HT.

The textural characteristics of the samples were examined through N_2_ physisorption. Figure 5 and Figure 6 depict the nitrogen sorption isotherms and pore size distribution curves (shown in the inset of the figures). The isotherms are classified as type IV according to the International Union of Pure and Applied Chemistry (IUPAC) classification [38], characterized by H3-type hysteresis loops, which indicate capillary condensation phenomena within mesoporous structures.

In general, mesoporosity can arise from the aggregation of primary HA nanoparticles resulting in inter-particle voids, or it can be an intra-particle porosity generated during the synthesis process. Analyzing the isotherms, it can be observed that in the case of the samples obtained by the hydrothermal method, the hysteresis loop appears at high values of the relative pressures, greater than 0.8, which suggests that the mesoporosity in this case is caused by the inter-particle voids generated by the agglomeration of HA nano-sized crystals. This hypothesis is also supported by the distribution of the pore sizes, which is very wide, extending over almost the entire range specific to mesopores (2–50 nm) and even exceeding it in the case of samples containing Sr in a percentage higher than or equal to 10%. Comparing the isotherms of the samples obtained by the two synthesis methods, it is obvious that the pore size distribution is broader in samples obtained hydrothermally than in those obtained by precipitation, whereas the average pore diameter is almost two times larger. The exception is the sample containing 30% Sr, for which both methods yield similar pore size distribution and mean pore diameter results. In the case of the samples obtained by precipitation, the values of the relative pressure at which the hysteresis loops close are lower than in the case of the hydrothermal method, around 0.7, which suggests that their porosity is mainly given by the inter-particle voids generated by aggregation of primary nanoparticles, but can also be attributed, to a lesser extent, to an intra-particle porosity.

As shown in Table 2, the specific surface areas of the samples obtained by precipitation are almost double compared to those obtained by the hydrothermal method, more precisely between 1.8 and 2 times larger, except for the sample containing 30% Sr, for which the ratio is 1.5. Total pore volumes of the samples obtained by precipitation are slightly higher compared to the corresponding ones obtained hydrothermally.

### 3.4. Scanning Electron Microscopy (SEM) Analysis

**Figure 7:**
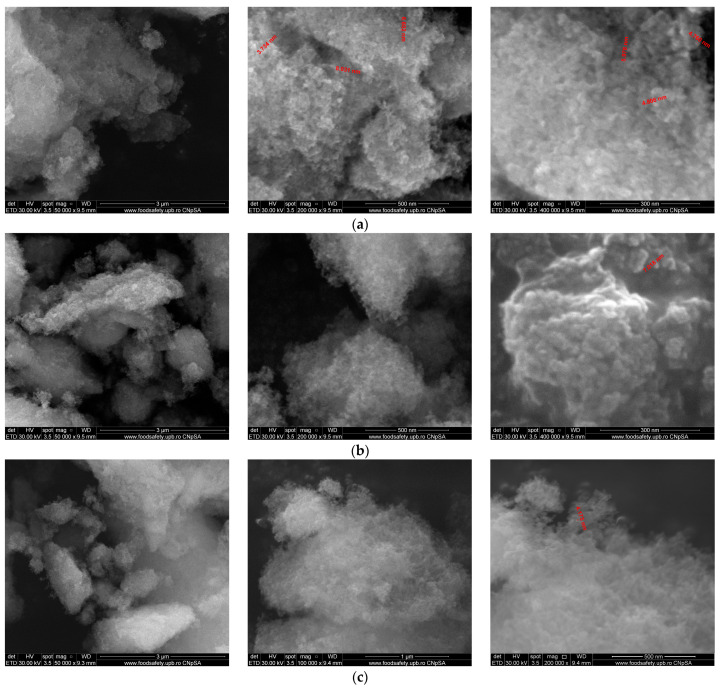

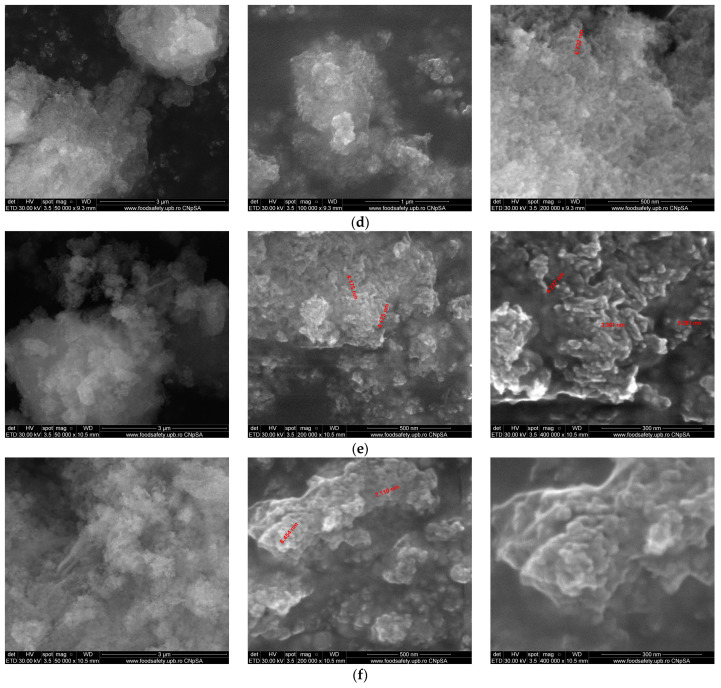
SEM images of HA powders synthesized by the precipitation method. (**a**)—HAPr, (**b**)—HAPr-Sr1%, (**c**)—HAPr-Sr5%, (**d**)—HAPr-Sr10%, (**e**)—HAPr-Sr20%, (**f**)—HAPr-Sr30%.

SEM was used to investigate the morphology and particle size of synthesized powders. The morphology of the NPs is an important characteristic that plays a role in controlling their interaction with proteins [39], and thus controlling the way biomaterial integrate in the host tissue. Typical SEM images of powders synthesized by the precipitation method are shown in Figure 7. The particles have a strong tendency to agglomerate and are difficult to distinguish in terms of size and shape. These morphological aspects are specific to HA powders synthesized by precipitation and could indicate the nano-size of the obtained HA particles.

**Figure 8:**
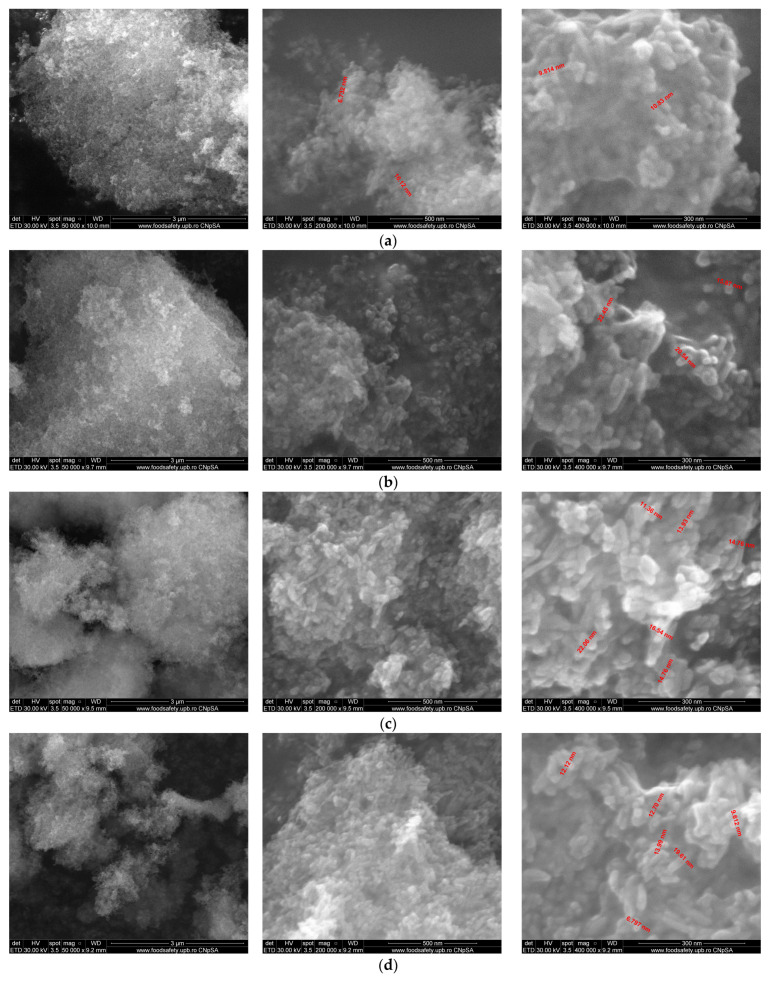

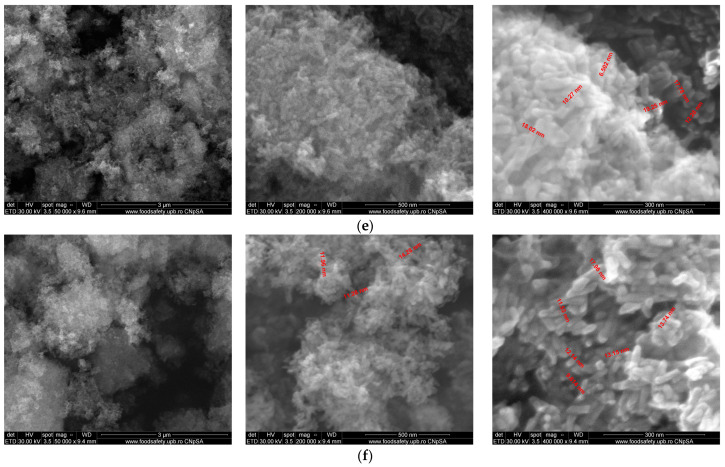
SEM images of HA powders synthesized by the hydrothermal method. (**a**)—HAHT, (**b**)—HAHT-Sr1%, (**c**)—HAHT-Sr5%, (**d**)—HAHT-Sr10%, (**e**)—HAHT-Sr20%, (**f**)—HAHT-Sr30%.

Larger and differently shaped particles were obtained through the hydrothermal method. Typical SEM images of powders synthesized by the hydrothermal method are shown in Figure 8.

### 3.5. Transmission Electron Microscopy (TEM) Analysis

**Figure 9:**
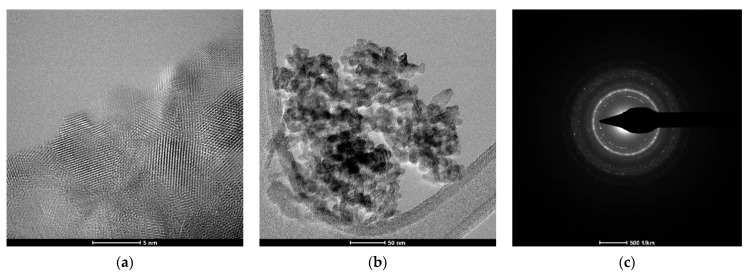
TEM images: (**a**,**b**), HAPR, (**c**) SAED image for HAPR.

**Figure 10:**
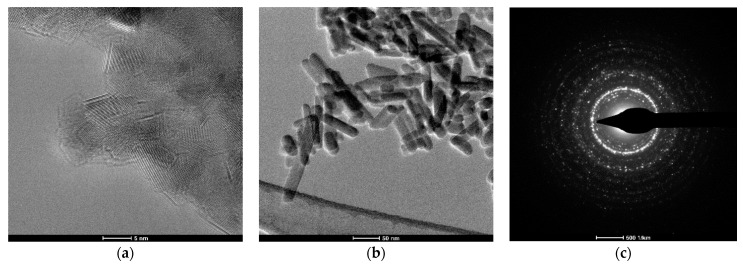
TEM images: (**a**,**b**), HAHT, (**c**) SAED image for HAHT.

TEM images show the nanostructure (Figure 9 and Figure 10) of HA/SrHA. It can be observed that the morphology of the nanoparticles is different depending on the synthesis route. Samples obtained by co-precipitation had a quasi-spherical shape, while the hydrothermally obtained samples had an elongated, rod-like shape. TEM images confirm the obtained nanoparticles’ agglomeration tendency. SAED images offer extra information on the crystallinity of the samples. The occurrence of diffraction rings with higher light intensity, in SAED images for HAHT, shows a higher degree of crystallinity compared with the HAPR sample.

**Figure 11:**
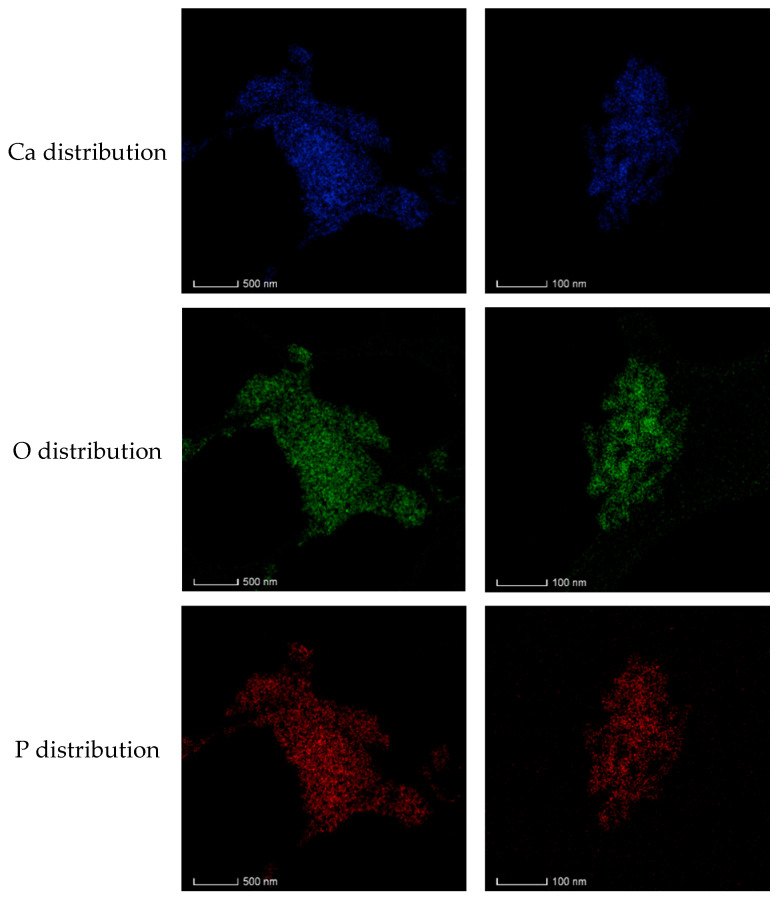

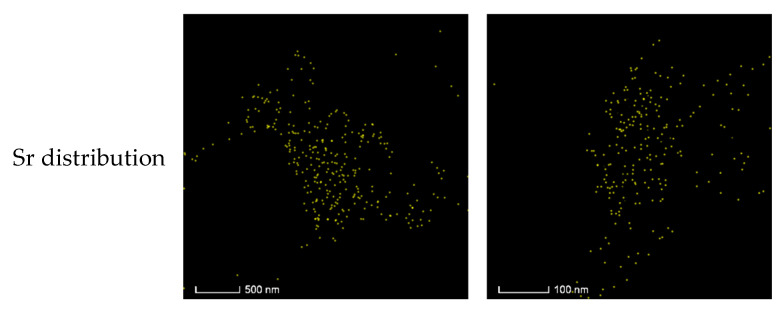
Electron energy loss spectroscopy (EELS) mapping of the HAHT-Sr1% sample showing the distribution of Ca, O, P, and Sr.

The elemental distribution mapping of samples confirms that Sr was incorporated into the matrix of SrHA. Figure 11 presents the corresponding electron energy loss spectroscopy (EELS) maps of the Ca, O, P, and Sr distribution for HAHT-Sr1%. All investigated elements had a homogeneous distribution even at a nanometric scale.

### 3.6. Energy Dispersive X-ray (EDX) Analysis

**Figure 12:**
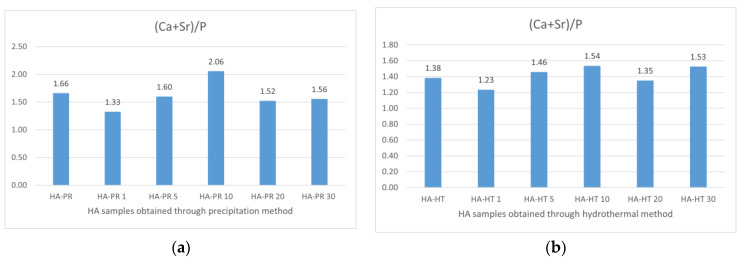
Calculated percent (Ca + Sr)/P for HA/SrHA obtained through the precipitation method (**a**) and the hydrothermal method (**b**).

**Figure 13:**
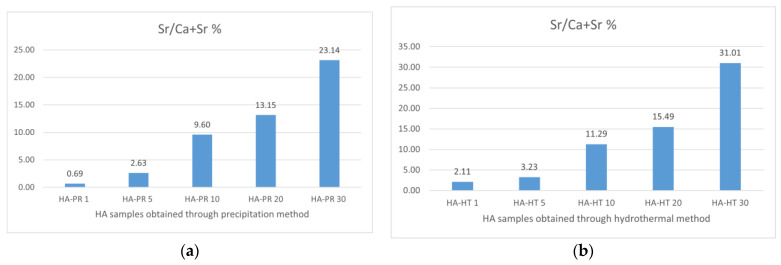
Calculated percent of Sr/(Ca + Sr) for HA/SrHA obtained through the precipitation method (**a**) and the hydrothermal method (**b**).

**Figure 14:**
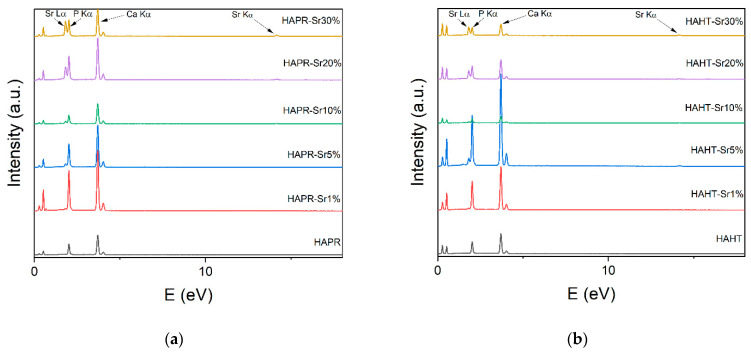
EDX analysis of HA/SrHA samples obtained through the precipitation method (**a**) and the hydrothermal method (**b**). The presence of HA-specific elements (Ca, P) can be observed, along with Sr.

Previous research has reported that biological apatite derived from bone products exhibits a Ca/P ratio ranging between 1.50 and 1.85, notably influenced by bone species and age. The contributing factors for this stoichiometry deviation are the cationic and anionic substitutions of Ca, PO_4_^3−^, or OH^−^ groups from the HA lattice with trace elements and CO_3_^2−^ or silicate groups. The calculated ratios of Ca/P and (Ca + Sr)/P, obtained through EDX for the synthesized samples (Figure 12 and Figure 13), differ from the theoretical one of 1.67 found in stoichiometric HA, but are similar to the ratio reported in the literature for synthesized HA and apatite provided from biogenic sources [40]. Also, the experimental Sr/(Ca + Sr) % ratio (Table 3) is different from the theoretical intended ratio, possibly due to the lesser accuracy of EDX in quantitative analysis, as mentioned by other authors [41]. The presence of Ca and Sr in the samples is highlighted by the peaks of the EDS spectrum (Figure 14).

### 3.7. Strontium-Ion Release by Inductively Coupled Plasma Atomic Mass Spectrometry Analysis (ICP-MS)

**Figure 15:**
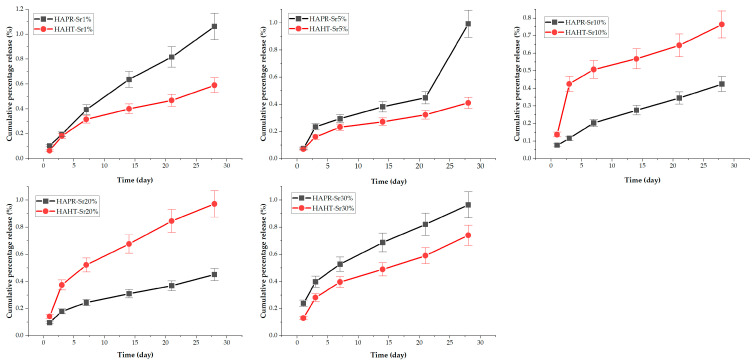
Cumulative percentage release of Sr from the synthesized samples at 37 °C and 200 rpm for different time intervals (1, 3, 7, 14, and 21 days).

In order to examine the release of Sr from prepared SrHA under dynamic conditions, powder samples were dispersed in PBS (10×) with a pH 7.4 at a concentration of 1.5 g/L. The preparation of the PBS was conducted in accordance with the established methodology provided by the producer (Roth). ICP results revealed different release patterns for Sr ions in the physiological medium during the 28 days of immersion, as shown in Figure 15. The concentrations of Sr ions released from the samples obtained by precipitation were generally higher than the concentrations of Sr ions released from samples of SrHA obtained by the hydrothermal method. Also, there was a steady increase in concentration of Sr ions for all the samples.

### 3.8. Cytotoxicity

**Figure 16:**
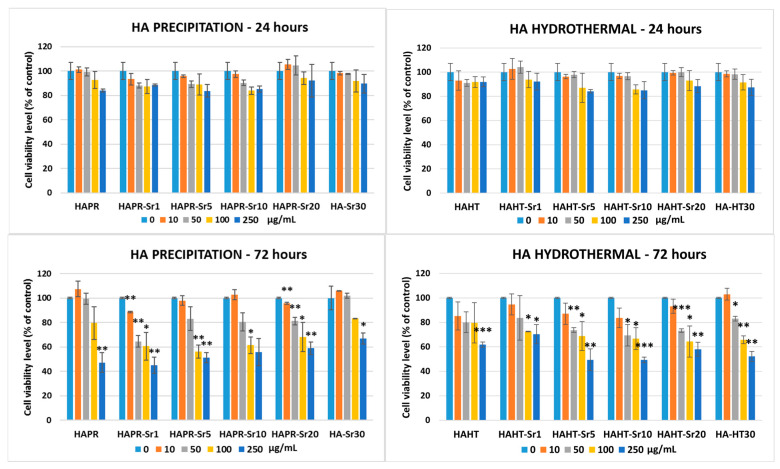
Cellular viability evaluation after 24- and 72-h incubation of MC3T3-E1 pre-osteoblasts with different concentrations of HA/SrHA samples. The results were calculated as mean ± SD (*n* = 3) and expressed relative to control cells (* *p* < 0.05, ** *p* < 0.01 and *** *p* < 0.001 compared to control).

The effect of various concentrations of HA/SrHA samples on cell viability was studied by the MTT assay; results are shown in Figure 16. Up to a concentration of 50 μg/mL of tested samples, no significant change was noticed in the number of viable cells after 24 h of incubation compared to the control. For this time exposure interval, a 15% decrease of control in cell viability was measured in the case of Sr 5% and Sr 10% samples. After 72 h, higher decreases in the viability of MC3T3-E1 pre-osteoblasts were obtained compared to the first period of exposure at concentrations higher than 10 μg/mL, resulting in 60% viability of controls or less in the case of 250 μg/mL. Overall, the samples obtained by precipitation showed better results after 72 h of incubation with 10 and 50 μg/mL compared to the other method of synthesis.

**Figure 17:**
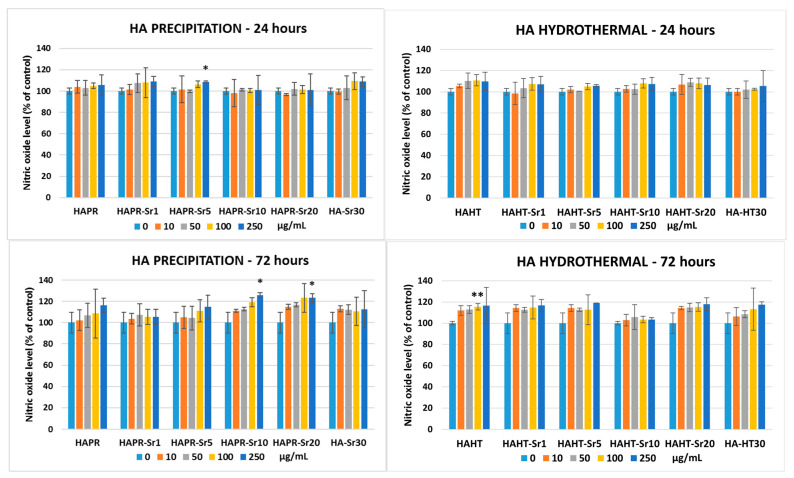
Nitric oxide level evaluation after 24- and 72-h incubation of MC3T3-E1 pre-osteoblasts with different concentrations of HA/SrHA samples. The results were calculated as mean ± SD (*n* = 3) and expressed relative to control cells (* *p* < 0.05 and ** *p* < 0.01 compared to control).

The amount of NO released in the culture medium was assessed as a useful indicator of inflammation and cell membrane damage after incubation with HA/SrHA. Figure 17 shows no changes higher than 10% and 20% increase in NO release after 24 and 72 h incubation, respectively, proving that these compounds are not potent inflammation inducers at the tested concentrations.

## 4. Conclusions

We synthesized HA/SrHA with different Sr molar ratio through precipitation and hydrothermal methods and thoroughly characterized them chemically, morphologically, and biologically. The purpose of incorporating Sr into SrHA is to access the benefits Sr has for bone regeneration, especially in the case of bones eroded by osteoporosis, while reducing or eliminating the systemic treatment with Sr compounds. This approach is achievable thanks to the versatility of the HA in terms of synthesis, cost, availability, and material composition.

As HA/SrHA obtained through the simple and cost-effective precipitation method resembles more the physico-chemical properties of bone mineral, having lower crystallinity, as well as carbonate and other cation/anion substitutions, it is recommended as a key biomaterial ingredient in future implants.

In general, the samples obtained through precipitation exhibited superior outcomes regarding cytotoxicity when compared to the hydrothermal synthesis method, but all samples showed low inflammation-inducing features at the tested concentrations, reconfirming the general potential of HA/SrHA in tissue engineering. On the longer exposure time, higher decreases in the viability of used pre-osteoblasts were observed compared to the shorter exposure period with higher concentration than 10 µg/mL. We are encouraged to believe that a lower concentration yields better results and that these materials may be used for the development of other biomaterials and implants for bone regeneration. More complex and patient-specific substitutes, including new composites using SrHA and innovative techniques like 3D printing, can better match the patient-specific needs of the native bone tissue.

## Figures and Tables

**Table 1:** Crystallite size, crystallinity, and lattice parameters of HA/SrHA samples.

Sample	2ϴ (002) (deg)	2ϴ (deg)/ (002) FWHM	Crystallite Size (002) (Å) Scher Eq	Χc	Lattice Parameters		V (Å^3^)
					a = b	c	
HAHT	25.75	0.416	205	0.19	9.4320	6.8899	530.82
HAHT-Sr1%	25.72	0.421	202	0.19	9.4372	6.8994	532.14
HAHT-Sr5%	25.67	0.424	201	0.18	9.4440	6.9081	533.12
HAHT-Sr10%	25.62	0.436	195	0.17	9.4593	6.9239	536.53
HAHT-Sr20%	25.44	0.458	186	0.14	9.4963	6.9662	544.04
HAHT-Sr30%	25.31	0.469	181	0.13	9.5288	7.0077	551.03
HAPR	25.76	0.561	152	0.08	9.4230	6.8841	529.36
HAPR-Sr1%	25.74	0.566	150	0.08	9.4317	6.8933	531.04
HAPR-Sr5%	25.68	0.565	151	0.08	9.4360	6.9024	532.23
HAPR-Sr10%	25.63	0.588	142	0.06	9.4651	6.9251	537.28
HAPR-Sr20%	25.48	0.581	146	0.07	9.5250	6.9650	547.24
HAPR-Sr30%	25.41	1.001	79	0.01	9.6001	7.0022	558.87
Hydroxyapatite—crystal system hexagonal; space group P63/m(176)-01-072-1243					9.4320	6.8810	530.14

**Table 2:** Textural parameters of the samples.

Sample	S_BET_ (m^2^g^−1^)	Total Pore Volume (cm^3^g^−1^)	Average Pore Size (nm)
HAPR	144.8	0.510	11.7
HAPR-Sr 1%	136.6	0.446	10.6
HAPR-Sr 5%	141.8	0.468	11.0
HAPR-Sr 10%	136.2	0.534	13.3
HAPR-Sr 20%	130.9	0.518	13.2
HAPR-Sr 30%	104.5	0.608	20.2
HAHT	73.3	0.479	22.2
HAHT-Sr 1%	74.3	0.450	21.0
HAHT-Sr 5%	74.5	0.470	21.4
HAHT-Sr 10%	75.9	0.502	23.5
HAHT-Sr 20%	71.3	0.460	23.3
HAHT-Sr 30%	68.7	0.458	23.7

**Table 3:** Element atomic presence in the obtained HA/SrHA samples.

Element Atomic %	HAPR	HAPR Sr1%	HAPR Sr5%	HAPR Sr10%	HAPR Sr20%	HAPR Sr30%	HAHT	HAHT Sr1%	HAHT Sr5%	HAHT Sr10%	HAHT Sr20%	HAHT Sr30%
C	0	3.38	1.07	11.9	0.36	8.15	48.97	24.49	9.8	55.48	51.77	55.86
O	69.6	73.74	68.68	59.47	67.9	65.38	42.47	60.92	69.85	38.22	41.6	39.39
P	11.42	9.83	11.63	9.37	12.57	10.35	3.59	6.53	8.28	2.48	2.82	1.88
Ca	18.98	12.96	18.13	17.42	16.64	12.39	4.97	7.89	11.68	3.38	3.22	1.98
Sr	0	0.09	0.49	1.85	2.52	3.73	0	0.17	0.39	0.43	0.59	0.89
Ca + Sr	18.98	13.05	18.62	19.27	19.16	16.12	4.97	8.06	12.07	3.81	3.81	2.87
(Ca + Sr)/P	1.66	1.33	1.60	2.06	1.52	1.56	1.38	1.23	1.46	1.54	1.35	1.53
Sr/(Ca + Sr) %	0.00	0.69	2.63	9.60	13.15	23.14	0.00	2.11	3.23	11.29	15.49	31.01

## Data Availability

Data are contained within the article.
